# Neuroinflammatory and morphological changes in late-life depression: the NIMROD study

**DOI:** 10.1192/bjp.bp.116.190165

**Published:** 2016-12

**Authors:** L. Su, Y. O. Faluyi, Y. T. Hong, T. D. Fryer, E. Mak, S. Gabel, L. Hayes, S. Soteriades, G. B. Williams, R. Arnold, L. Passamonti, P. Vázquez Rodríguez, A. Surendranathan, R. W. Bevan-Jones, J. Coles, F. Aigbirhio, J. B. Rowe, J. T. O'Brien

**Affiliations:** Li Su, PhD, **Yetunde O. Faluyi**, MBChB, Department of Psychiatry, University of Cambridge, UK; **Young T. Hong**, PhD, **Tim D. Fryer**, PhD, Wolfson Brain Imaging Centre and Department of Clinical Neurosciences, University of Cambridge, UK; **Elijah Mak**, BA, Department of Psychiatry, University of Cambridge, UK; **Silvy Gabel**, MSc, Department of Psychiatry, University of Cambridge, UK and Faculty of Psychology and Neuroscience, Maastricht University, the Netherlands; **Lawrence Hayes**, MBBS, **Soteris Soteriades**, BA, Department of Psychiatry, University of Cambridge, UK; **Guy B. Williams**, PhD, Wolfson Brain Imaging Centre and Department of Clinical Neurosciences, University of Cambridge, UK; **Robert Arnold**, BSc, Department of Psychiatry, University of Cambridge, UK; **Luca Passamonti**, MD, **Patricia Vázquez Rodríguez**, MSc, Department of Clinical Neurosciences, University of Cambridge, UK, **Ajenthan Surendranathan**, MRCP, **Richard W. Bevan-Jones**, MBBChir, Department of Psychiatry, University of Cambridge, UK; **Jonathan Coles**, PhD, Division of Anaesthesia, Department of Medicine, University of Cambridge, UK; **Franklin Aigbirhio**, DPhil, Wolfson Brain Imaging Centre and Department of Clinical Neurosciences, University of Cambridge, UK; **James B. Rowe**, PhD, Department of Clinical Neurosciences, University of Cambridge and Medical Research Council, Cognition and Brain Sciences Unit, Cambridge, UK; **John T. O'Brien**, DM, Department of Psychiatry, University of Cambridge, UK

## Abstract

We studied neuroinflammation in individuals with late-life depression, as a risk factor for dementia, using [^11^C]PK11195 positron emission tomography (PET). Five older participants with major depression and 13 controls underwent PET and multimodal 3T magnetic resonance imaging (MRI), with blood taken to measure C-reactive protein (CRP). We found significantly higher CRP levels in those with late-life depression and raised [^11^C]PK11195 binding compared with controls in brain regions associated with depression, including subgenual anterior cingulate cortex, and significant hippocampal subfield atrophy in cornu ammonis 1 and subiculum. Our findings suggest neuroinflammation requires further investigation in late-life depression, both as a possible aetiological factor and a potential therapeutic target.

Late-life depression is known to be associated with specific clinical features, such as cognitive impairments, it typically has a poor outcome and is a risk factor for dementia. Vascular factors have been implicated in its aetiology,^1^ but neuroinflammation has not been well studied despite being a highly plausible mechanism and potentially tractable target. We have previously shown an increase in inflammatory cytokines in the blood in older individuals with depression.^2^ In the current study we aimed to show whether we could demonstrate an increase in central (brain) inflammation *in vivo* using [^11^C]PK11195 positron emission tomography (PET) imaging. [^11^C]PK11195 is a radioligand that selectively binds to the translocator protein (TSPO), a receptor expressed on activated microglia. Increased binding has been found in stroke, traumatic brain injury and some neurodegenerative diseases, such as Alzheimer's disease.^3^ We also investigated vascular and structural changes in late-life depression using multimodal magnetic resonance imaging (MRI).

## Method

Within the Neuroimaging of Inflammation in Memory and Other Disorders (NIMROD) study, we recruited five participants with depression aged 65–78 years (depression group) from secondary care National Health Service (NHS) psychiatry services, who had met DSM-IV criteria for major depression (assessed using the Structured Clinical Interview for DSM Disorders)^4^ and 13 controls (59–81 years) from the NIHR Clinical Research Network. Participants had full clinical and cognitive assessment and venepuncture for C-reactive protein (CRP) measurement. All participants provided written informed consent. Ethics approval for the study protocol was obtained from the National Research Ethics Service – East of England Committee.

Participants underwent multimodal MRI on a 3T Siemens Verio scanner including *T*_1_ weighted structural (176 slices, 1 × 1 mm, 1 mm slice thickness, reaction time (TR) = 2300 ms, echo time (TE) = 2.98 ms, flip angle 9), *T*_2_ FLAIR (75 slices, 0.9 × 0.9 mm, 2 mm slice thickness, TR = 12540 ms, TE = 132 ms, flip angle 120) and high-resolution hippocampal *T*_2_ coronal (24 slices, 0.4 × 0.4 mm, 2 mm slice thickness, TR = 6420 ms, TE = 11 ms, flip angle 160). Within 4 months of MRI scans, PET imaging was performed on a GE Advance scanner (GE Healthcare, Waukesha, Wisconsin) for 75 min following bolus intravenous injection of [^11^C]PK11195 (500 MBq), with a pre-injection 15 min ^68^Ge/^68^Ga transmission scan used for attenuation correction.

To estimate TSPO binding site density, non-displaceable binding potential (BP_ND_) was determined from [^11^C]PK11195 PET data with the guidance of *T*_1_ weighted MRI.^5^ Regional white matter hyperintensity (WMH) volumes were segmented and quantified using *T*_2_ FLAIR images. *T*_2_ hippocampal coronal scans were manually segmented for cornu ammonis 1 (CA1), CA2, CA3/dentate gyrus, and measured for subiculum and entorhinal thickness. (See online supplement DS1 for detailed methods of PET analysis as well as WMH and hippocampal segmentation.)

As a result of the relatively small sample size in the depression group, we did not assume Gaussian data distributions. Group-level statistical comparisons of regional [^11^C]PK11195 PET BP_ND_ as well as demographic, cognitive, blood, white matter lesion and hippocampal subfields data were performed using non-parametric Mann–Whitney *U*-test. Chi-squared test was used to test for gender differences between groups. In addition, regional [^11^C]PK11195 BP_ND_ data were also analysed using Monte Carlo randomisation tests to obtain *P*-values for each participant. (See online supplement DS1 for further details.) Results are reported without correction for multiple comparisons, noting that the primary outcome measure related to our principal hypothesis was [^11^C]PK11195 BP_ND_ in limbic cortical regions associated with depression and dementia.

## Results

The two groups did not differ in age, gender ratio, education or global cognition (Mini-Mental State score^6^) but the depression group had significantly higher blood CRP levels than controls (mean CRP: depression group 18.8 mg/L, control group 1.2 mg/L; *P* = 0.002) and a trend in Montgomery–Åsberg Depression Rating Scale^7^ score (depression group 10.0, control group 4.0; *W* = 13.5, *P* = 0.065).

**Fig. 1:**
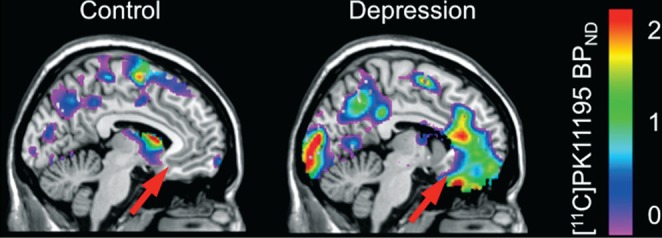
Statistical comparison of individual participant's [^11^C]PK11195 non-displaceable binding potential (BP_ND_) with the control group (*Z*-scores). Red arrow: subgenual anterior cingulate cortex.

Although largely recovered from their depression at time of imaging, at the group level, participants with depression had significantly higher [^11^C]PK11195 BP_ND_ compared with controls in left subgenual anterior cingulate cortex (mean BP_ND_: depression group 0.1103, control group 0.0246; *W* = 54, *P* = 0.035), and right parahippocampus (depression group 0.1225, control group 0.0490; *W* = 53, *P* = 0.046); these are substantiated by the voxel-wise results given in Fig. 1 and online Fig. DS5. Using the individual-level Monte Carlo randomisation test, all five individuals in the depression group showed a significant increase of [^11^C]PK11195 BP_ND_ in the aforementioned brain regions, confirming the group-level statistical inference.

The depression group showed trends for more extensive WMH in both periventricular (depression group 7.33 ml, control group 3.74 ml; *W* = 13, *P* = 0.059) and deep (depression group 1.85 ml, control group 0.73 ml; *W* = 15, *P* = 0.095) white matter. We found a significant reduction of CA1 area in the coronal plane (depression group 22.09 mm^2^, control group 24.90 mm^2^; *W* = 52, *P* = 0.019) and subiculum thickness (depression group 1.73 mm, control group 1.95 mm; *W* = 56, *P* = 0.004) in the depression group. (See online supplement DS2 for additional demographic, cognitive, WMH and volumetric results.)

## Discussion

We found evidence of both central and peripheral inflammation in older individuals with depression, including changes in the anterior cingulate and medial temporal lobe, which play a key role in the regulation of mood and cognitive functioning.^8^ Damage in these areas is linked with an elevated risk of dementia.^9^ Increased [^11^C]PK11195 binding in people with depression could be associated with cerebrovascular disease and white matter lesions, reported in the current and previous studies,^10^ although some controls also had a similar burden of WMH with normal levels of [^11^C]PK11195 binding in subgenual anterior cingulate cortex and parahippocampus (online Fig. DS5). It is notable that [^11^C]PK11195 BP_ND_ showed the greatest effect size compared with other modalities, with a 300% increase from controls (*v.* 150% for WMH and 10% for hippocampal atrophy), suggesting a strong biomarker potential for late-life depression.

There was no major cognitive impairment in our cohort, although the depression group showed significant atrophy in the hippocampus and subiculum, which have been shown to correlate with greater risk of cognitive impairment and Alzheimer's disease.^11^ In addition, the hippocampus is a key component in the hypothalamic–pituitary–adrenal (HPA) axis. Increases in cytokine levels can lead to increases in oxidative stress and glucocorticoid as well as decreases in serotonin and other neurotransmitters in HPA resulting in impaired mood and cognition.^12,13^

Our results were not corrected for multiple comparisons, and further replication is required in a larger cohort. However, the large effect size of [^11^C]PK11195 was in keeping with our principal hypothesis and was supported by both a primary group-level test and secondary individual statistical tests. Cross-sectional studies provide limited information about whether neuroinflammation was the cause or consequence of neuronal damage in affected brain areas, so future longitudinal studies are needed. In conclusion, we suggest that neuroinflammation may be an important mechanism in late-life depression and merits further investigation as a potential target for novel therapeutics in a condition that responds poorly to conventional antidepressant therapy.
